# Time of Dietary Energy and Nutrient Intake and Body Mass Index in Children: Compositional Data Analysis from the Childhood Obesity Project (CHOP) Trial

**DOI:** 10.3390/nu14204356

**Published:** 2022-10-18

**Authors:** Vanessa Jaeger, Berthold Koletzko, Veronica Luque, Mariona Gispert-Llauradó, Dariusz Gruszfeld, Piotr Socha, Elvira Verduci, Gian Vincenzo Zuccotti, Louise Etienne, Veit Grote

**Affiliations:** 1 Division of Metabolic and Nutritional Medicine, Department of Pediatrics, Dr. von Hauner Children’s Hospital, University Hospital, LMU Munich, 80337 Munich, Germany; 2 Paediatrics Research Unit, Universitat Rovira I Virgili-IISPV, 43201 Reus, Spain; 3 Serra Hunter Fellow, Universitat Rovira I Virgili-IISPV, 43201 Reus, Spain; 4 Neonatal Department and Neonatal Intensive Care Unit, Children’s Memorial Health Institute, 04-730 Warsaw, Poland; 5 Department of Gastroenterology, Hepatology, Nutritional Disorders and Pediatrics, Children’s Memorial Health Institute, 04-730 Warsaw, Poland; 6 Department of Paediatrics, Vittore Buzzi Children’s Hospital, University of Milan, 2012 Milan, Italy; 7 Groupe Santé CHC, Bd. Patience et Beaujonc 2—(B), 4000 Liège, Belgium

**Keywords:** chrono-nutrition, overweight, eating pattern, compositional data analysis, children, macronutrients, energy, meal timing

## Abstract

Meal timing is suggested to influence the obesity risk in children. Our aim was to analyse the effect of energy and nutrient distributions at eating occasions (EO), including breakfast, lunch, supper, and snacks, on the BMI z-score (zBMI) during childhood in 729 healthy children. BMI and three-day dietary protocols were obtained at 3, 4, 5, 6, and 8 years of age, and dietary data were analysed as the percentage of the mean total energy intake (TEI; %E). Intakes at EOs were transformed via an isometric log–ratio transformation and added as exposure variables to linear mixed-effects models. Stratified analyses by country and recategorization of EOs by adding intake from snacks to respective meals for further analyses were performed. The exclusion of subjects with less than three observations and the exclusion of subjects who skipped one EO or consumed 5% energy or less at one EO were examined in sensitivity analyses. Around 23% of the children were overweight at a given time point. Overweight and normal-weight children showed different distributions of dietary intakes over the day; overweight children consumed higher intakes at lunch and lower intakes of snacks. However, no significant effects of timing of EOs on zBMI were found in regression analyses.

## 1. Introduction

Childhood obesity is a major public health concern with detrimental consequences for health and wellbeing [1,2] whereby diet is a decisive and modifiable factor contributing to obesity. Childhood represents an age when food preferences and dietary patterns develop, and hence, it is a time window with particular preventive potential [3,4].

Increasingly, it has been reported that not only what and how much is eaten, but also when food is eaten may matter. Chrono-nutrition refers to this hypothesis and is based on the interaction between biological rhythms, temporal eating patterns, and its influence on metabolic health [5,6]. The hypothesis is driven by the assumption that food intake at different times of the day may have beneficial or detrimental effects on weight and other health conditions [7,8]. In particular, higher food intake at supper [9] or evening/night snacks have been associated with adverse effects on weight status in children [10,11], whereas the contribution of breakfast to TEI was reported to have neutral effects [12]. Higher food intake at lunch was associated with a lower overweight risk [13]. Only limited data from studies in children and adolescents investigating the effects of the time of macronutrient intake are available. One study found that lower proportions of nutrients in the afternoon and evening are associated with a lower body weight [10], whereas another study found that low fat and high carbohydrate (CHO) intake in the morning are associated with a higher body fat mass index [14].

A systematic review by Almoosawi et al. [7] examined the effects of the time of day of energy intake and obesity and concluded that further studies are needed to establish a potential association. Specifically, studies are needed which examine simultaneously all EOs throughout the day, as other EOs may affect the intake at one EO. Previous studies examined different EOs throughout the day but analyzed only one EO at a time with respect to being overweight/obese in regression analyses [12], probably due to collinearity issues between EOs. Thus, we propose compositional data analysis, which is widely used in other disciplines (e.g., geology) but is rarely used in nutritional epidemiology [15]. Compositional data analysis is specifically designed to simultaneously analyze the effects of different parts of a total (here: parts are intakes at EOs which add to the TEI) on an outcome. A further asset of compositional data analysis is the possibility to disentangle the effects of meal timing from the total energy or nutrient intake, and thus, may reveal estimates that are more accurate. By compositional data analysis, we are able to examine the effect of meal timing by shifting energy or energy from nutrients from one EO to another. For instance, for a given TEI, how is the BMI affected if more energy is distributed to supper and less to the remaining EOs?

The aims of the following study are, firstly, to describe the time-of-day energy and energy from nutrient intakes (CHO, protein, and fat) in relation to being overweight in European children aged 3 to 8 years. Secondly, we aim to examine the effect of meal timing by analyzing the (re-) distribution of energy and energy from nutrients throughout the day (breakfast, lunch, supper, and snacks) on zBMI in a longitudinal study.

## 2. Materials and Methods

### 2.1. Study Design and Population

This analysis was based on data from the Childhood Obesity Project Trial, a randomized controlled trial that recruited 1678 healthy, full-term infants in the first 8 weeks of life from 2002 to 2004. The aim of the intervention was to examine the effect of different protein content in infant formula consumed during the first year of life on later obesity risk [16,17]. An observational arm of breastfed children was also included in the study. The infants were recruited in Belgium, Germany, Italy, Poland, and Spain. Legal guardians of the participating infants provided written informed consent prior to study enrollment. For the analysis of this paper, data from the follow-up visits performed during 2005 to 2012 at the ages of 3, 4, 5, 6, and 8 years were used. The trial was conducted in accordance with the Declaration of Helsinki, approved by the ethical committee from each study site, and registered at clinicaltrials.gov (NCT00338689).

### 2.2. Dietary Assessment

Parents or caretakers provided weighted and estimated dietary records over three consecutive days (2 weekdays and 1 weekend day). Food and leftovers were weighed using food scales (Unica 66006; Soehnle, Murrhardt, Germany). If weighing foods was not possible, an atlas with food pictures was used to support estimations of portion sizes.

Dietary records were entered in a database specifically created for this study, with data mainly based on the German food composition database BLS 2.3 (Bundeslebensmittelschlüssel). When necessary, the database was enriched by information from product labels, manufacturers, or national food databases of participating countries. Nutritional values of products were updated to BLS 3.01 for analysis. Trained dieticians discussed open issues in protocols with participants and executed quality checks of the collected dietary records following standard operating procedures.

An EO is defined as any occasion where food or beverages are consumed. Predefined categories with typical country-specific time slots were used for this study to enter protocol data according to the following EOs: breakfast, lunch, and supper for meals as well as morning, afternoon, and evening for snacks. Country-specific differences in times of EOs between weekdays and weekends were considered.

The misreporting of energy intake was calculated by comparing individual cut-offs with the ratio of reported energy intake to estimated energy requirements [18]. Dietary records were assumed to be implausible when the individual ratio fell below or above these cut-offs, but were not excluded from the analysis as previously recommended [19].

### 2.3. Anthropometric Measurement (Outcome)

At each time point, weight and height were measured twice while wearing only light clothes following standard operating procedures based on the WHO growth reference study [20]. Measurements were performed using the same weight scale (SECA 702) and stadiometer (SECA 242) at each study site. Body mass index (BMI = weight (kg)/height (m)²) was calculated and standardized for age and sex (zBMI) according to the WHO reference population [20,21]. Children were classified as overweight or obese according to the cut-offs defined by the International Obesity Task Force (IOTF) [22]. The IOTF, in contrast to the WHO, provide a coherent cut-off for the entire observation period from 3 to 8 years.

### 2.4. Statistical Analysis

Anthropometric and/or dietary data were obtained at 3, 4, 5, 6, and 8 years of age. All subjects with measurements for both anthropometric and dietary data were added from each observation time point. From the total number of observations, all children with at least one out of five observation time points were included in the analysis. TEI and energy intake at each EO were calculated as the mean daily intake (kcal/day) of all valid protocol days. Proportional intakes (%E) at EOs were calculated as the respective intake (kcal) of the EO towards TEI. Data are presented as arithmetic means (standard deviation) for continuous variables and as counts (%) for categorical variables. Compositional data are displayed with geometric means and a variation matrix.

Meal timing was analyzed by compositional data analysis. Compositional data analysis applied to health research is described elsewhere [23,24,25]. Briefly, the basis of compositional data analysis is that several parts of a total are examined concurrently. In this study, the parts are the proportional intakes at EOs in which parts add to 100% of the mean daily TEI and total CHO, protein, and fat intake, respectively, recorded at a particular age and study visit. Furthermore, the total sum (here: TEI or total CHO, protein, and fat intake, respectively, as 100% or 1) is fixed, which is practical as we do not want to investigate the increase in TEI or nutrient intake on BMI as in usual regression analyses. Instead, we want to investigate the shift in intake at EOs through the day for a given TEI or nutrient intake. Both the fixed sum and the collinearity between parts imply that the compositional data need to be transformed to be used in linear regression analysis. Firstly, the ratio between parts needs to be calculated. This is performed by sequential binary partitioning (SBP), which splits parts of a composition step-by-step and in a hierarchical manner in smaller groups. The SBP for each EO at first rank is displayed in Table 1.

Only the first partition includes the relevant information to examine the effect of the EO of interest, however the other partitions are needed to include the co-dependence between parts. Secondly, all partitions are transformed into new variables (coordinates) via isometric log–ratio (ILR) transformations. The rationale and the process of how to perform compositional data analysis are described in detail in the Appendix A.

The ILR coordinates are calculated by means of logarithm. This implies that the not consumed EOs (so-called zero values) of any dietary record have to be either discarded (the complete dietary record) or the EOs with zero values need to be adapted. Most of the zero values were present in snacks, especially during evenings. Therefore, intakes from snacks during the morning, afternoon, and evening were amalgamated to one EO (snacks). For the remaining zero values (*n* = 39) the parametric robust expectation maximisation algorithm was applied as recommended [26]. Zero values were replaced for each observation time point without distorting the ratio between parts.

Linear mixed-effects models were used to estimate the effects of different distributions of energy and macronutrient intakes throughout the day on zBMI. Mixed-effects models enable the estimation of variables with repeated measurements and allow for missing values at observation time points. Individual regressions were estimated for each set of ILR coordinates (exposure) with the zBMI as the outcome. Each regression contained subject-specific random intercepts and random slopes over age with a piecewise linear spline (knot at 6 years). Relevant covariates were chosen by backwards selection and likelihood ratio testing. This resulted in parental BMI, country, TEI, misreporting as recommended [27], and an interaction term between TEI and country. Further covariates were considered (smoking during pregnancy, sex, intervention type, and parental education), but none of those increased the model fit. Two sub-analyses for each EO were carried out. Firstly, analyses were stratified by country to better account for differences in daytime of the meals. Secondly, EOs were recategorized to examine snack intake in a time-dependent way. For this, the intake from snacks were added to the respective intake from meals and the new EOs resulted in morning (breakfast + morning snack), afternoon (lunch + afternoon snack), and evening (supper + evening snack).

The recategorized EOs were used to calculate “eveningness” as termed by Diederichs et al. [28]. “Eveningness” was calculated as the difference between evening and morning intake for energy and energy from nutrients. However, we expressed “eveningness” in percentage points (pp):

“Eveningness” (pp) = intake at evening from total intake (%)—intake at morning from total intake (%)

The analyses were further evaluated by performing sensitivity analyses including only subjects with at least 3 out of 5 observation time points and by the exclusion of subjects who skipped an EO or consumed very little at one EO (EO with 5% of the TEI or less). Residuals and influential observations were checked graphically, and a few influential observations were removed from the analysis (*n* = 13). All analyses were performed using RStudio [29] version 4.04 in addition with the statistical packages “lme4”, “robCompositions”, and “zCompositions”. Results were considered as significant if *p* < 0.05.

## 3. Results

**Figure 1:**
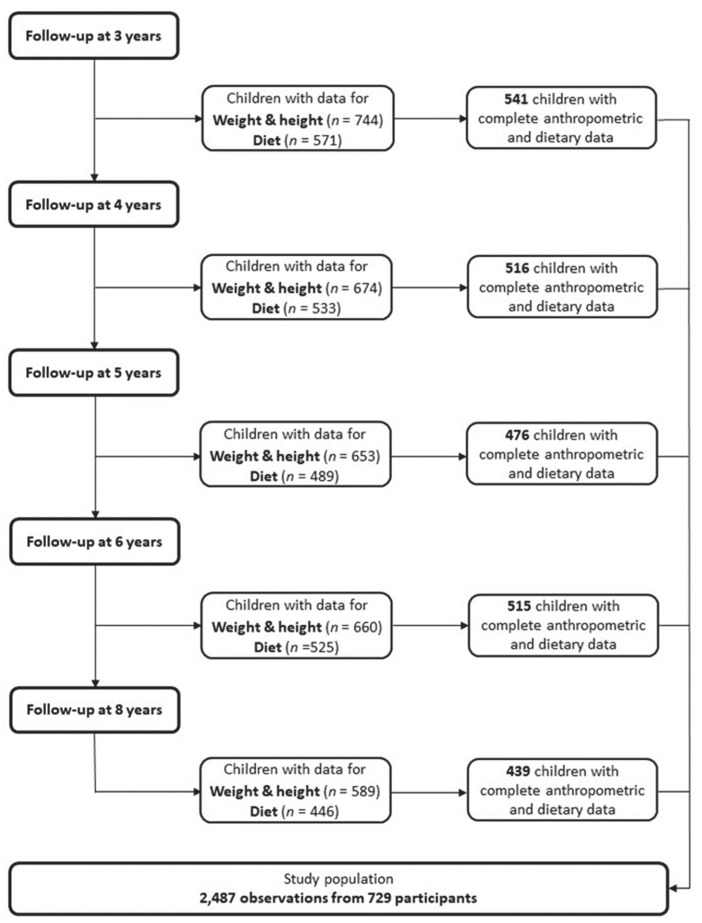
Number of participating children with available data for anthropometric and/or dietary data obtained at 3, 4, 5, 6, and 8 years of age. Children with measurements for both anthropometric and dietary data were added from each observation time point (*n* = 2487), resulting in 729 children.

Data were available for 729 healthy children (53% girls) with 2487 observations (Figure 1). Most children were recruited in Spain (29%), followed by Italy (27%), Poland (17%), Germany (14%), and Belgium (13%). Children participated on average in 3.4 out of 5 follow-up visits, with 32% of children participating in all 5 follow-up visits. The portion of children who participated in the majority of visits (more than 3 visits) was lower if their parents had a low education level. Half of the parents whose children participated in this study have an intermediate level of education. Dietary records were provided for a mean of 2.9 days. In total, 13% of all records were classified as under-reported and 12% of the records as over-reported. The dietary records of overweight children were more often under-reported (37% in overweight children, 9% in normal-weight children) and less often over-reported than the dietary records of normal-weight children (3% in overweight children, 13% in normal-weight children). In 45% of the children, at least one parent was overweight or obese, and in 20% of the children, both parents were overweight or obese. The highest number of overweight children as a percentage of the total number of children in each country were seen in Italy and Spain (18% each), followed by Poland (15%). Smoking during pregnancy was observed in 28% of all children with the highest numbers in Poland and Spain (34% each).

Table 2 describes the study population with anthropometric and dietary data by age. The children in this study population were slightly heavier than the WHO reference population (zBMI > 0). Approximately 23% of the children were overweight, and around 6% of the children were obese (highest at 6 years: 6%, lowest at 4 years: 2%) at a given time point. Food intake (kcal/day) increased with age, but food intake in relation to body weight (kcal/kg/day) declined with age. The composition of food intake (CHO, fat, and protein) remained constant with age.

The variations at EOs were estimated as a variation matrix, with values close to 0 indicating a high proportionality/co-dependence between ratios. The highest proportionality was observed between lunch and supper, and the lowest proportionality was observed between snacks and supper for energy and energy from nutrients (Supplementary Material—Table S5). The average eating frequency per day (foods and beverages excluding water and unsweetened tea) declined from 5.8 to 5.1, while the average frequency of snacks declined from 3.1 to 2.3 from 3 to 8 years of age. The number of children with an average snack frequency of more than three snacks per day decreased from 21% to 5% in the same age range.

**Figure 2:**
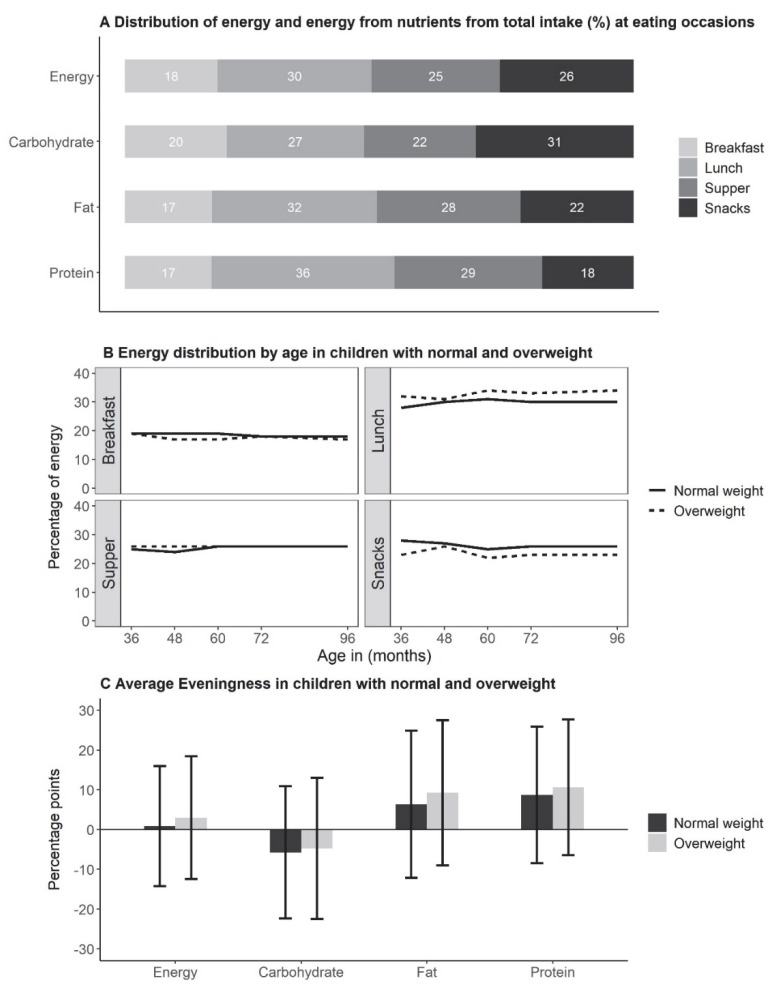
Distribution of energy and macronutrients by eating occasion, evening preference (“eveningness”), time, and overweight status of 729 children. (**A**) Average distribution of energy and nutrient intake as a percentage of the total intake at eating occasions; (**B**) Average energy intake at each eating occasion and age stratified by weight status. (**C**) Average eveningness ^§^ in percentage points (pp) stratified by weight status (mean ± standard deviation). ^§^ Difference in percentage intake between evening (supper + evening snack) and morning (breakfast + morning snack). Weight status was defined for normal-weight and overweight children according to IOTF. All analyses were performed in children aged 3 to 8 years (overweight children: n_observations_ = 366, normal-weight children: n_observations_ = 2121).

The distribution of energy and nutrient intakes according to EOs averaged by age is shown in Figure 2A. Most energy intakes were consumed at lunch, followed by snacks, supper, and breakfast. Protein and fat intakes were mostly consumed at lunch, followed by supper, whereas most of CHO intakes were consumed with snacks. Overweight children consumed higher intakes of total energy, protein, and fat than normal-weight children and consumed proportionally higher energy intakes at lunch and less at snacks (Figure 2B). Similar differences in the proportional intakes at EOs between overweight and normal-weight children were seen for energy from nutrients.

If the intake from snacks were added to the intake from respective meals with the EO categories morning, afternoon, and evening, most foods were consumed in the afternoon (46%E ± 9), followed by the evening (27%E ± 9) and morning (26%E ± 8). The intakes for energy from protein and fat followed a similar distribution, whereas energy intakes for CHO were larger in the morning (29%E) compared to the evening (24%E). A higher “eveningness” (difference between evening and morning intake) was observed for protein and fat intakes, whereas energy intakes were similar, and CHO intakes showed a negative “eveningness”. However, the variations in energy and energy from nutrients were large in both weight groups (Figure 2C).

The results of the regression analysis on the effects of meal timing, expressed by the distribution of energy and energy from nutrients over the day in EOs on zBMI, are depicted in Table 3. The interpretation of the β-estimates estimated by compositional data analysis is different from usual regression analyses. The compositional data analysis split the exposure variable into co-dependent parts that are interpreted concurrently, whereas in usual regressions, only one exposure variable is interpreted at a time. Specifically, the ratio between EOs is interpreted. The β-estimates of the ILR coordinates applied to our results are interpreted as follows: For a given TEI, the redistribution of energy intake with an increase in energy at breakfast as compared to the other EOs was not significantly associated with zBMI (β = −0.02; *p* > 0.05). Similarly, the redistribution of energy with an increase at lunch, supper, or snacks compared to the remaining EOs and a given TEI were not significantly associated with zBMI (*p* > 0.05). Furthermore, results of different distributions of energy from nutrients throughout EOs were not statistically significant, as well as all sensitivity analyses (Supplementary Materials Tables S1 and S2). Stratification by country and reorganizing EOs to examine snacks in a time-dependent manner revealed no significant effects (Supplementary Materials Tables S3 and S4).

## 4. Discussion

In this large study evaluating the data from 729 healthy children aged 3 to 8 years from five European countries, children who are overweight consumed more energy than children with normal weights and also had a different intake distribution during the day, with higher intakes at lunch and fewer intakes of snacks. Despite these differences between normal-weight and overweight children, no statistically significant differences in weight status were seen for intakes with any EO. Thus, the distribution of energy and macronutrient intake over the day had no significant impact on weight status in children.

We found overweight children to consume proportionally lower intakes with snacks and higher intakes with lunch than normal-weight children. A similar pattern was also seen in French children aged 3–6 years and 7–11 years, but with significant differences in snack and main meal contribution only in older children [12]. A possible explanation for this pattern in overweight children might be that access to snacks is limited and/or consumption of snack foods is restricted due to parental concerns, as reported previously [30]. It has been suggested that healthy snacks, defined as nutrient dense and with a low energy density, during the mid-morning and mid-afternoon promote appetite control and may prevent overeating at the following meal [31,32]. However, shifting more energy or energy from nutrients to snacks and less to meals did not affect zBMI in children, as implied by our regression analyses.

The timing of food intake and its distribution throughout the day have been examined in this study, without appreciable effects of timing of energy or macronutrient intakes on BMI in children. Some studies found associations of meal timing with weight status in children [10,11,13,33], but another study did not [34]. Comparisons with other studies are difficult since we used an entirely different methodological approach. Furthermore, no generally agreed definition exists on how to determine a meal or a snack [7,30,35], which further limits comparisons.

Our study population exhibited a regular eating pattern where the skipping of meals was rarely seen, as previously shown [36]. A systematic review suggested that the skipping of meals, especially breakfast, is negatively associated with being overweight in children [37]. Meal skipping was also negatively associated with children’s diet quality [38,39]. Thus, the more regular eating patterns of our study population may have contributed to the absence of an association of EO timing with BMI.

The chronotype of individuals might influence the timing of food intake. A chronotype is defined as an individual’s circadian preference/phenotype and is classified as a morning or evening chronotype [8]. Studies in children are limited, but studies in adolescents and adults showed that individuals with an evening chronotype prefer to eat later in the day [40]. Although we have not assessed the chronotype of the study participants, our results showed a similar evening and morning ratio for energy, which might indicate that few children exhibit an evening chronotype. The chronotype in children generally tends to be early and shifts to later times in adolescence [41]. An evening chronotype was associated with more frequent breakfast skipping [40] or less fruit and vegetable consumption [42], both being associated with a higher weight status [43]. Thus, it is possible that the relatively low degree of “eveningness” in our study could have influenced our results.

Randomized controlled clinical trials and observational studies in overweight adults have shown the timing of food intake to modify weight-loss effectiveness. For instance, overweight and obese women have lost significantly more weight if an evening meal was consumed at earlier compared to later times [44] and if lunch was consumed earlier compared to later [45]. Possible mechanisms which may explain the timing of food intake as a risk factor for being overweight or weight-loss effectiveness are circadian rhythms [5,6]. Briefly, the circadian timing system is responsible for daily biological rhythms and synchronizes physiological and behavioural aspects to the outside world. External cues, such as the light–dark cycle or timing of food intake, provide signals to entrain the circadian clocks. It has been suggested that the timing of food intake aligned to the circadian rhythms of metabolic processes may be beneficial for health. For instance, it has been shown that insulin and glucose exhibit a circadian rhythm, with a decrease in insulin sensitivity and glucose tolerance during the day and a nadir in the evening [46,47]. A further factor might be diet-induced thermogenesis, which is not only affected by the nutrient composition of meals but was also found to be higher in the morning than in the evening [48,49]. These factors might contribute to potential untoward effects of higher evening intakes on weight status in adults, whereas no such evidence is available in children.

The present study benefited from the longitudinal study design with five years of follow-up, which allowed for examining between and within differences in a large number of study participants. Furthermore, the inclusion of children from five European countries enhances the generalizability of our study findings and the use of 3-day weighted dietary records allowed for a more accurate description of dietary intake than other methods. Another strength of our study is the applied compositional data analysis by taking into account EOs of an entire day concurrently. In addition, compositional data analysis has the benefit to disentangle the effects of meal timing from TEI, which allowed for a comprehensive isocaloric analysis. These results add to the limited understanding between meal timing and overweight in children.

One of the limitations of this study is that EOs were analyzed in categories (breakfast, lunch, supper, and snacks) which allowed for comparing and analysing EOs between countries. However, the timing of EOs may vary between countries, which could not be considered in the analysis. We tried to counteract the limitation by stratified analyses for each country. Another limitation is that we could not perform a detailed analysis of snack intakes by time of day because many children did not consume snacks, particularly in the evening, but also during mornings. However, we examined snacks in a time-related manner by summarizing meals and snacks combined into morning, afternoon, and evening. A further limitation of this study is a potential social desirability bias during data collection, suggested by the lower snack intake and the lower number of possible under-reporting of TEI in overweight compared to normal-weight children. The social desirability bias describes a behaviour in which an individual reports selected foods or beverages due to societal beliefs or norms to act in favour of the researcher [50]. We tried to minimize this bias by including misreporting as a covariate in all regression analyses.

Further studies examining the relationship between meal timing and obesity might also take the chronotype of each child into consideration. Studies in adults have shown that the chronotype influenced the effect between meal timing and being overweight [51]. The development of consistent and broadly agreed definitions on how to define a meal and a snack would be most helpful to enable comparisons of the respective results from different studies and to strengthen conclusions.

## 5. Conclusions

This study described the diurnal differences in energy and nutrient intakes in overweight compared to normal-weight children, with proportionally lower reported intakes at snacks and higher intakes at lunch in overweight children. The timing of meals and snacks did not influence the weight status in the total population of children. These data do not provide a basis for approaches to reduce the obesity risk in children based on the shifting distribution of dietary intakes across EOs.

## Figures and Tables

**Table 1:** Sequential binary partitioning of a four-part composition (breakfast, lunch, supper, and snacks) with each eating occasion at first rank.

Partition	Breakfast	Lunch	Supper	Snacks
1	Breakfast: mean (lunch, supper, snacks)	Lunch: mean (supper, snacks, breakfast)	Supper: mean (snacks, breakfast, lunch)	Snacks: mean (breakfast, lunch, supper)
2	Lunch: mean (supper, snacks)	Supper: mean (snacks, breakfast)	Snacks: mean (breakfast, lunch)	Breakfast: mean (lunch, supper)
3	Supper: Snacks	Snacks: Breakfast	Breakfast: Lunch	Lunch: Supper

**Table 2:** Description of study population from age 3 to 8 years.

Age in Years	3 (*n* = 541)	4 (*n* = 516)	5 (*n* = 476)	6 (*n* = 515)	8 (*n* = 439)	Overall (*N* = 729) *
BMI (kg/m^2^)	16.0 ± 1.3	15.9 ± 1.4	15.9 ± 1.7	16.0 ± 2.0	16.9 ± 2.7	16.1 ± 1.9
zBMI	0.3 ± 1.0	0.4 ± 1.0	0.4 ± 1.0	0.4 ± 1.2	0.4 ± 1.2	0.4 ± 1.1
Overweight **, N *** (%)	52 (9.6)	62 (12)	68 (14.3)	85 (16.5)	99 (22.6)	366 (14.7)
Energy (kcal/day)	1202 ± 237	1307 ± 235	1374 ± 249	1454 ± 250	1568 ± 285	1374 ± 279
kcal/kg/day	82.5 ± 17.7	78.2 ± 16.0	72.0 ± 15.7	67.2 ± 14.0	56.6 ± 13.5	71.9 ± 17.9
Carbohydrate (%E)	50.4 ± 7.7	50.0 ± 7.0	50.2 ± 7.2	50.3 ± 6.9	49.0 ± 7.0	50.0 ± 7.2
g/day	149.2 ± 44.3	160.1 ± 35.8	171.2 ± 43.5	183.0 ± 43.3	192.2 ± 49.0	170.3 ± 45.8
g/kg/day	10.3 ± 3.4	9.6 ± 2.4	9.0 ± 2.6	8.5 ± 2.3	6.9 ± 2.1	8.9 ± 2.8
Protein (%E)	15.3 ± 3.0	15.0 ± 2.9	14.9 ± 2.9	14.9 ± 2.6	15.2 ± 2.8	15.0 ± 2.8
g/day	44.9 ± 11.6	48.0 ± 12.3	50.5 ± 12.8	54.0 ± 12.5	59.5 ± 14.6	51.1 ± 13.6
g/kg/day	3.1 ± 0.8	2.9 ± 0.8	2.6 ± 0.7	2.5 ± 0.6	2.1 ± 0.6	2.7 ± 0.8
Total fat (%E)	34.3 ± 6.2	34.9 ± 5.8	34.9 ± 5.7	34.8 ± 5.6	35.8 ± 5.8	34.9 ± 5.8
g/day	45.0 ± 12.4	50.1 ± 13.1	52.8 ± 13.4	56.3 ± 13.6	62.7 ± 16.6	53.0 ± 15.0
g/kg/day	3.1 ± 0.9	3.0 ± 0.8	2.8 ± 0.8	2.6 ± 0.7	2.3 ± 0.7	2.8 ± 0.8

Values are presented as arithmetic mean ± standard deviation or as otherwise indicated. * In total, 729 subjects with 2487 observations. ** According to the cut-offs from the International Obesity Task Force [22]. *** Number of observations. Abbreviations: BMI = body mass index; zBMI = body mass index z-score; %E = energy from nutrients in percentage from total energy intake.

**Table 3:** Regression results of ILR coordinates against body mass index z-score from age 3 to 8 years (*N* = 729 children).

ILR *	Energy			Carbohydrate			Protein			Fat		
	β	SE	*p*-Value	β	SE	*p*-Value	β	SE	*p*-Value	β	SE	*p*-Value
*Breakfast*	−0.02	0.02	0.429	−0.01	0.02	0.493	0.00	0.02	0.957	−0.02	0.02	0.192
*Lunch*	0.00	0.03	0.945	0.01	0.02	0.747	0.00	0.02	0.942	0.00	0.02	0.825
*Supper*	0.01	0.03	0.616	−0.00	0.02	0.864	−0.01	0.02	0.577	0.03	0.02	0.123
*Snacks*	0.01	0.02	0.677	0.01	0.02	0.580	0.01	0.02	0.472	−0.01	0.01	0.380

Estimates were based on linear mixed-effects models which contained a subject-specific random intercept and a slope for age. The random slope is estimated by piecewise linear splines with a knot at 6 years. The analysis was adjusted for each set of ILR coordinates, parental BMI, misreporting, country, total energy intake, and interaction between country and total energy intake. * ILR coordinates refer to the mentioned eating occasion in relation to the geometric mean of the remaining eating occasions. Abbreviation: SE—standard error.

## Data Availability

Data available on request due to ethical restrictions.

## Supplementary Materials

The following supporting information can be downloaded at: https://www.mdpi.com/article/10.3390/nu14204356/s1, Table S1: Regression results of ILR coordinates of EOs against body mass index z-score including only subjects with at least three observation time points; Table S2: Regression of ILR coordinates against body mass index z-score excluding subjects with intakes of 5% or less of the total intake at an eating occasion; Table S3: Regression of ILR coordinates against body mass index z-score stratified by country; Table S4: Regression of ILR coordinates for morning (breakfast + morning snack), afternoon (lunch + afternoon snack), evening (supper + night snack), and ratio of evening and morning intake (“eveningness”) against body mass index z-score; Table S5: Mean and pair-wise variation of eating occasions for energy and energy from nutrient intake in children aged 3 to 8 years.

## Abbreviations

BMI—body mass index; CHO—carbohydrates; EO—eating occasion; ILR—isometric log ratios; TEI—total energy intake; WHO—World Health Organization; zBMI—body mass index z-score.

## Appendix A

### Compositional Data Analysis and Calculation of ILR-Coordinates

Dietary data are typical examples of compositional data because the contribution of dietary nutrients depends on each other, and they sum to the TEI. Nutrients are not consumed isolated, and a variation in one nutrient likely leads to variations in further nutrients. Likewise, the size of a meal depends on the size of the previous meal and/or if a snack was consumed between meals. This correlation between nutrients or meals induces collinearity between parts when analyzed as independent variables in regression analyses simultaneously. Due to the collinearity between independent variables, it is difficult to measure the influence of individual variables on the outcome which may lead to unstable and inaccurate coefficients [23].

Compositional data is best analyzed by expressing the composition with a set of logarithmized ratios (log ratio) [52]. The log ratio approach is an established method in many scientific disciplines (e.g., geology). Dumuid et al. [24,53], Corrêa Leite et al. [15], and Chastin et al. [25] have described in detail the log ratio approach for use in health research and nutritional epidemiology. Briefly, a composition is a set of parts which sum to a whole. For instance, energy intake at breakfast, lunch, supper, and snacks are a composition of four parts and sum to the TEI. The composition is analyzed as a ratio, which highlights the relative nature of the parts (each part as a proportion of the TEI). Consequently, if one part of a composition increases, the other part(s) need to decrease to keep the sum constant. This dependence between parts and the constant sum assumption imply that the composition is constrained, but regression analyses are typically conducted in unconstrained real space in which each part can vary freely. Thus, the composition needs to be transformed into real space, which is performed using a specific set of log ratios, namely the isometric log ratio (ILR) coordinates. The derivation of the ILR was described elsewhere [15,24].

Compositional data analysis in the present study has the following properties: Firstly, it is possible to analyze all EOs simultaneously without biasing the coefficient due to collinearity. Secondly, the transformed variables of interest are independent from the TEI and enable us to disentangle the effect of meal timing from the effect of the TEI. Thirdly, the generic effect of the TEI on the outcome is considered. Further methods in nutritional epidemiology are available to deal with appropriate considerations of the TEI, such as the residual method [54]. However, to our knowledge, none of these methods include appropriate adjustments of the TEI and additionally deal with perfect multi-collinearity.

The ILR coordinates were calculated based on an orthonormal basis. A widely used approach to generate the basis is sequential binary partitioning (SBP) [55], which splits parts of a composition step-by-step and in a hierarchical manner into smaller groups. An example of a SBP with breakfast at first rank is given in Table A1. In a first step, the composition is split in two groups that will form the parts of the ratio. Parts, which belong to the first group, are coded as 1, and parts which belong to the second group are coded as −1. This leads to the first partition. In a second step, one of the two groups in step one is further split into two new groups. The parts, which belong to the first group, are coded as 1, and parts which belong to the second group are coded as −1. Parts, which do not belong to one group at that order are coded as 0. The second step results in the second partition. This procedure is continued until a subgroup consists only of one part. Formally, a composition D is split in (D-1) parts.

**Table A1:** Sequential binary partition of a four-part composition with breakfast at first rank.

Partition	Breakfast	Lunch	Supper	Snacks	Ratio
1	1	−1	−1	−1	Breakfast: mean (lunch, supper, snacks)
2	0	1	−1	−1	Lunch: mean (supper, snacks)
3	0	0	1	−1	Supper: Snacks

1: Part is assigned to the first group; −1: part is assigned to the second group; 0: part is not involved in the partition at that order.

The partition is in the next step transformed into an ILR coordinate for each of the (D-1)-dimensional partitions presented using the following formula [52]:

$${\mathrm{ILR}}_{i}=\frac{\sqrt{\left(r*s\right)}}{\sqrt{\left(r+s\right)}}\ln\left(\frac{y_{i}}{g\left(y_{j}\right)}\right),\;for\;i=1,\;\ldots ,\;D-1$$

- *r* and *s*, respectively, are the number of parts in the first (coded as 1) and second group (coded as −1) at each order of the partition;
- $y_{i}$ is the proportional intake (coded as 1);
- $g(y_{j})\;$ is the geometric mean of the components of *y*, for *j* = *i* + 1,…, D (geometric mean of the components coded as −1).

We used an SBP in which the first partition group consist of only one (so-called pivot coordinate [56]). The advantage of pivot coordinates is that the first coordinate contains all the relevant information of the first part (here: breakfast) compared to the remaining parts which facilitate interpretation. All remaining coordinates are included in the regression analysis as covariates to consider the co-dependence between EOs. Inference of the other parts of the composition (lunch, supper, and snacks) is received by changing the hierarchical order of the composition. Thus, SBP was conducted four times, with each time a different EO at first rank, and each SBP resulted in three pivot coordinates. A set of pivot coordinates was defined as consisting of one SBP with resulting coordinates.
