# Supplementary material for: Time of Dietary Energy and Nutrient Intake and Body Mass Index in Children: Compositional Data Analysis from the Childhood Obesity Project (CHOP) Trial
Source: Nutrients. 2022 Oct 18;14(20):4356. doi: 10.3390/nu14204356 (PMC9610148; doi:10.3390/nu14204356)
Supplement: Supplementary file 1 [file nutrients-14-04356-s001.zip › nutrients-1923613-supplementary/Supplementary material-Table S1.pdf]

Supplementary material – Results of sensitivity analyses

*Table S1: Regression results of ILR coordinates of EO against body mass index z-score including only subjects with at least three observation time points (N = 514)**

ILR**	Energy			Carbohydrate			Protein			Fat		
	β	SE	p-value	β	SE	p-value	β	SE	p-value	β	SE	p-value
**Breakfast**	-0.03	0.03	0.179	-0.03	0.02	0.185	-0.01	0.02	0.576	-0.03	0.02	0.132
**Lunch**	-0.02	0.03	0.558	-0.01	0.02	0.724	-0.01	0.03	0.686	0.00	0.02	0.862
**Super**	0.03	0.03	0.228	0.02	0.02	0.462	0.00	0.02	0.928	0.03	0.02	0.095
**Snacks**	0.02	0.02	0.374	0.02	0.02	0.269	0.03	0.02	0.162	-0.01	0.01	0.490

Estimates were based on linear mixed effects models, which contained a subject-specific random intercept and slope for age. The random slope is estimated by piecewise linear splines with a knot at 6 years. Each analysis is adjusted for set of ILR coordinates, parental BMI, misreporting, country, total energy intake and interaction between country and total energy intake. * Results of 514 subjects with 2,160 observations. ** ILR coordinates are referring to the mentioned eating occasion in relation to geometric mean of remaining eating occasions. Abbreviation: SE – Standard error.
