# Supplementary material for: Time of Dietary Energy and Nutrient Intake and Body Mass Index in Children: Compositional Data Analysis from the Childhood Obesity Project (CHOP) Trial
Source: Nutrients. 2022 Oct 18;14(20):4356. doi: 10.3390/nu14204356 (PMC9610148; doi:10.3390/nu14204356)
Supplement: Supplementary file 1 [file nutrients-14-04356-s001.zip › nutrients-1923613-supplementary/Supplementary material-Table S2.pdf]

Supplementary material – Results of sensitivity analyses

*Table S2: Regression of ILR coordinates against body mass index z-score excluding subjects with intake of 5% or less of total intake at an eating occasion (N = 699)**

ILR**	Energy			Carbohydrate			Protein			Fat		
	β	SE	p-value	β	SE	p-value	β	SE	p-value	β	SE	p-value
**Breakfast**	-0.01	0.04	0.736	-0.02	0.03	0.486	0.03	0.03	0.416	-0.01	0.03	0.746
**Lunch**	-0.02	0.04	0.556	0.01	0.03	0.717	-0.03	0.03	0.314	-0.02	0.03	0.492
**Super**	0.01	0.04	0.860	-0.02	0.03	0.503	-0.03	0.03	0.371	0.02	0.02	0.481
**Snacks**	0.03	0.03	0.353	0.03	0.03	0.306	0.03	0.03	0.232	0.01	0.02	0.691

Estimates were based on linear mixed effects models, which contained a subject-specific random intercept and slope for age. The random slope is estimated by piecewise linear splines with a knot at 6 years. Analysis adjusted for set of ILR coordinates, parental BMI, misreporting, country, total energy intake and interaction between country and total energy intake. *Results of 699 subjects with 2,098 observations in total. **ILR coordinates are referring to the mentioned eating occasion in relation to geometric mean of remaining eating occasions. Abbreviation: SE – Standard error.
