# Supplementary material for: Time of Dietary Energy and Nutrient Intake and Body Mass Index in Children: Compositional Data Analysis from the Childhood Obesity Project (CHOP) Trial
Source: Nutrients. 2022 Oct 18;14(20):4356. doi: 10.3390/nu14204356 (PMC9610148; doi:10.3390/nu14204356)
Supplement: Supplementary file 1 [file nutrients-14-04356-s001.zip › nutrients-1923613-supplementary/Supplementary material-Table S3.pdf]

Supplementary material - Results of secondary analyses and energy distribution at eating occasions

*Table S3: Regression of ILR coordinates against body mass index z-score stratified by country (Italy: N = 196; Spain: N = 208; Poland: N = 126; Belgium: N= 97; Germany: N = 102)**

ILR**	Breakfast			Lunch			Supper			Snacks		
	β	SE	p-value	β	SE	p-value	β	SE	p-value	β	SE	p-value
**Energy**												
**Belgium**	-0.08	0.06	0.195	-0.06	0.08	0.440	0.08	0.07	0.225	0.05	0.06	0.354
**Germany**	-0.04	0.07	0.612	0.02	0.07	0.727	0.05	0.07	0.488	-0.04	0.06	0.517
**Italy**	-0.05	0.04	0.227	0.10	0.05	0.066	-0.04	0.05	0.394	-0.01	0.03	0.789
**Poland**	0.14	0.10	0.156	-0.10	0.09	0.265	0.02	0.08	0.767	-0.06	0.08	0.436
**Spain**	0.04	0.04	0.403	-0.04	0.06	0.461	-0.03	0.05	0.570	0.04	0.05	0.421
**Carbohydrate**												
**Belgium**	-0.10	0.05	0.055	-0.04	0.06	0.535	0.07	0.06	0.295	0.07	0.06	0.192
**Germany**	0.00	0.07	0.956	-0.01	0.06	0.892	0.01	0.07	0.853	-0.01	0.06	0.879
**Italy**	-0.02	0.03	0.549	0.05	0.04	0.200	-0.02	0.04	0.627	-0.02	0.03	0.557
**Poland**	0.04	0.08	0.587	0.01	0.08	0.886	-0.05	0.07	0.503	-0.01	0.08	0.902
**Spain**	0.01	0.04	0.839	0.01	0.05	0.782	-0.02	0.03	0.576	0.00	0.04	0.965
**Protein**												
**Belgium**	-0.03	0.05	0.568	-0.10	0.06	0.096	0.09	0.06	0.130	0.05	0.05	0.318
**Germany**	-0.02	0.07	0.726	0.08	0.06	0.132	0.01	0.06	0.914	-0.07	0.05	0.142
**Italy**	-0.03	0.03	0.422	0.06	0.05	0.198	-0.05	0.04	0.185	0.02	0.03	0.372
**Poland**	0.12	0.08	0.165	-0.12	0.08	0.133	0.04	0.07	0.544	-0.04	0.06	0.508
**Spain**	0.05	0.05	0.306	0.00	0.05	0.941	-0.07	0.04	0.110	0.03	0.04	0.513
**Fat**												
**Belgium**	-0.04	0.04	0.344	-0.01	0.05	0.885	0.06	0.04	0.208	-0.01	0.03	0.739
**Germany**	-0.03	0.04	0.466	0.00	0.04	0.979	0.06	0.05	0.173	-0.03	0.03	0.363
**Italy**	-0.05	0.03	0.082	0.07	0.04	0.073	-0.01	0.04	0.747	-0.01	0.02	0.598
**Poland**	0.09	0.06	0.161	-0.07	0.07	0.306	0.07	0.06	0.229	-0.09	0.05	0.101

**Spain**	0.02	0.03	0.467	-0.06	0.04	0.146	-0.01	0.04	0.694	0.05	0.03	0.134

Estimates were based on linear mixed effects models, which contained a subject-specific random intercept and slope for age. For Italy and Spain, the random slope is estimated by piecewise linear splines with a knot at 6 years. Analysis adjusted for set of ILR coordinates, parental BMI, misreporting and total energy. *Belgium: 97 children with 305 observations; Germany: 102 children with 299 observations; Italy: 196 children with 767 observations; Poland: 126 children with 401 observations and Spain: 208 children with 703 observations. **ILR coordinates are referring to the mentioned eating occasion in relation to geometric mean of remaining eating occasions. Abbreviation: SE – Standard error.
