# Supplementary material for: Time of Dietary Energy and Nutrient Intake and Body Mass Index in Children: Compositional Data Analysis from the Childhood Obesity Project (CHOP) Trial
Source: Nutrients. 2022 Oct 18;14(20):4356. doi: 10.3390/nu14204356 (PMC9610148; doi:10.3390/nu14204356)
Supplement: Supplementary file 1 [file nutrients-14-04356-s001.zip › nutrients-1923613-supplementary/Supplementary material-Table S4.pdf]

Supplementary material - Results of secondary analyses and energy distribution at eating occasions

Table S4: Regression of ILR coordinates for Morning (Breakfast + Morning snack), Afternoon (Lunch + Afternoon snack), Evening (Supper + Night snack) and ratio of Evening and Morning intake ("Eveningness") against body mass index z-score (N = 729)

ILR	Energy			Carbohydrate			Protein			Fat		
	β	SE	p-value	β	SE	p-value	β	SE	p-value	β	SE	p-value
**Morning***	-0.02	0.03	0.553	-0.01	0.03	0.830	0.00	0.03	0.942	-0.03	0.02	0.182
**Afternoon***	-0.01	0.03	0.761	-0.00	0.03	0.952	-0.01	0.03	0.756	-0.01	0.02	0.686
**Evening***	0.03	0.03	0.347	0.01	0.02	0.754	0.01	0.02	0.785	0.04	0.02	0.077

Estimates were based on linear mixed effects models, which contained a subject-specific random intercept and slope for age. The random slope is estimated by piecewise linear splines with a knot at 6 years. Analysis adjusted for set of ILR coordinates, parental BMI, misreporting, country, total energy intake and interaction between country and total energy intake. *ILR coordinates are referring to the mentioned eating occasion in relation to geometric mean of remaining eating occasions. Abbreviation: SE – Standard error.
