# Supplementary material for: Time of Dietary Energy and Nutrient Intake and Body Mass Index in Children: Compositional Data Analysis from the Childhood Obesity Project (CHOP) Trial
Source: Nutrients. 2022 Oct 18;14(20):4356. doi: 10.3390/nu14204356 (PMC9610148; doi:10.3390/nu14204356)
Supplement: Supplementary file 1 [file nutrients-14-04356-s001.zip › nutrients-1923613-supplementary/Supplementary material-Table S5.pdf]

Supplementary material – Variation matrix

Table S5: Mean and pair-wise variation** of eating occasions for energy and energy from nutrient intake in children aged 3 to 8 years (N = 729)*

	Breakfast	Lunch	Supper	Snacks
**Energy**				
**Geometric mean (%)**	17	29	24	23
**Variation matrix: Breakfast**	0.00	0.20	0.23	0.29
**Lunch**		0.00	0.13	0.29
**Supper**			0.00	0.51
**Snacks**				0.00
**Carbohydrate**				
**Geometric mean (%)**	18	26	20	28
**Variation matrix: Breakfast**	0.00	0.27	0.32	0.28
**Lunch**		0.00	0.21	0.32
**Supper**			0.00	0.59
**Snacks**				0.00
**Protein**				
**Geometric mean (%)**	15	34	27	15
**Variation matrix: Breakfast**	0.00	0.28	0.35	0.48
**Lunch**		0.00	0.16	0.48
**Supper**			0.00	0.81
**Snacks**				0.00
**Fat**				
**Geometric mean (%)**	15	30	26	18
**Variation matrix: Breakfast**	0.00	0.39	0.44	0.57
**Lunch**		0.00	0.25	0.50
**Supper**			0.00	0.85
**Snacks**				0.00

* The sum of the respective geometric means of each eating occasions do not sum to 100%;**Variation of logarithms of ratios (log-ratios). Values of ratios close to zero indicate high proportionality.
